# What is Ethical Accountability?

**DOI:** 10.1017/jme.2025.10194

**Published:** 2025

**Authors:** Melissa M. Goldstein

**Affiliations:** Department of Health Policy and Management, The George Washington University Milken Institute School of Public Health, United States

**Keywords:** ethical accountability, public health ethics, AI Governance

## Abstract

Farman Saeed Sedeeq and Percem Arman’s article aims to develop a framework of AI governance that avoids shortcomings in existing models such as limited enforceability and rigid data-sharing rules. The goal of the weighty undertaking is to develop a “structured yet flexible approach” to balancing AI advancements in public health with ethical imperatives. Three core “pillars” are used for evaluation: ethical accountability, regulatory adaptability, and transparency. The concept of ethical accountability is explored briefly in this commentary.

Farman Saeed Sedeeq and Percem Arman’s article in this issue aims to develop a framework of AI governance that avoids shortcomings in existing models such as limited enforceability and rigid data-sharing rules. The goal of the weighty undertaking is to develop a “structured yet flexible approach” to balancing AI advancements in public health with ethical imperatives. Three core “pillars” are used for evaluation: ethical accountability, regulatory adaptability, and transparency.^1^ It is the concept of ethical accountability that is explored briefly here.

Sedeeq and Arman explain the idea in part by discussing algorithmic bias, which (according to the authors) persists because of unrepresentative training data and leads to disparities in diagnostic accuracy across demographic groups. Weak accountability frameworks, such as the WHO’s ethical guidelines, fail to mandate corrective measures or redress mechanisms in such cases.^2^ Instead, ethical accountability in AI-driven public health should demand rigorous bias audits, corrective measures such as dataset diversification, and redress mechanisms for affected populations. Accountability mechanisms should also enforce equitable resource allocation and AI innovation, requiring accessibility audits and infrastructure investments in underserved regions.^3^ Elsewhere in the article, Sedeeq and Arman point to a mechanism where the framework would require developers to obtain certifications prior to deploying AI systems in healthcare and would be subject to penalties “including fines of up to 2% of global review” for violations.^4^ Details on issues such as redress mechanisms, enforcement, and penalty assessments are scarce.

Disparities in diagnostic accuracy, barriers to AI-enabled healthcare in underserved regions, transparency, and explainability standards all deserve robust public policy debate and should be addressed clearly in AI governance models. It is less clear whether AI’s integration into health systems introduces *new* dimensions of ethical responsibility in public health or whether methods of addressing such issues in governance models (or even law) are rightly considered *ethical* accountability.

Accountability, defined generally as “an obligation or willingness to accept responsibility or to account for one’s actions,”^5^ could imply many types of responsibilities in AI governance. Critical questions abound, such as how accountability standards should be set, who will decide whether standards have been met, who should be held accountable in what cases, and to whom an individual or organization might be held accountable. In addition, appropriate redress mechanisms, enforcement, and penalty assessments (if any) must be defined and roles and responsibility for their appropriate adjudication and imposition must be delineated. While these types of discussions might be common in law and public policy, it is questionable whether they are the norm, or even achievable, in ethics discourse.

An example of this divergence can be found in the discussion of algorithmic bias noted above. Sedeeq and Arman state that “racially biased referral algorithms have systematically underestimated the care needs of Black patients, reflecting historical inequities embedded in training data.”^6^ The framework’s approach to ethical accountability, in this case, “requires governance frameworks to mandate” the corrective measures and redress mechanisms noted above.^7^

The *ethics* principles and theories implicated by racially biased referral algorithms involve equity and justice. The principles of autonomy and fidelity also become relevant in a discussion that includes transparency, explainability, and personal choice for the population involved. While these principles are not explored fully in the article, Sedeeq and Arman do note that the systematic review they conducted identified three “interconnected ethical priorities,” including “mitigating bias, ensuring accessibility and upholding informed consent.”^8^ Of course, some of the disconnect noted herein could reflect definitional variation. Still, it is unclear that rigorous bias audits, dataset diversification, redress mechanisms, or penalty assessments address *violation of ethics principles*, as opposed to poorly constructed algorithms and computer models.

In their seminal article, “Public Health Ethics: Mapping the Terrain,” a prominent group of ethicists describes a set of general moral considerations relevant to public health. Public health ethics, they note, “involves ongoing efforts to specify and to assign weights to these general moral considerations in the context of particular policies, practices, and actions, in order to provide concrete moral guidance.”^9^ The principles noted above (equity, justice, autonomy, fidelity) are part of this group. The authors also discuss “a process-oriented approach” to public accountability, which involves gathering input from the populations involved in such policies and providing justifications for them.^10^ Public accountability, in this model, ensures that trade-offs between individual interests and collective needs will be made openly and acknowledges that people’s fundamental well-being and values are at stake. Accountability “requires that reasons, grounded in ethics, will be provided to those affected by the decisions. It provides a basis for public trust, even when policies infringe or appear to infringe on some general moral considerations.”^11^

Sedeeq and Arman’s undertaking is commendable in its breadth and effort to promote equitable public health outcomes. The article recognizes that the ethical implications of AI in healthcare are critical to consider as we develop responsive governance frameworks and proposes ideas for addressing the difficulty of developing enforcement, accountability, and redress mechanisms in law and governance. While *ethical accountability* may not precisely describe the public health process we seek, *public accountability*, as described by Childress et al., does require that we incorporate general moral considerations as we deliberate appropriate public health policies, practices, and actions. As they note, this idea of accountability is both prospective and retrospective, and is critical for building and sustaining public trust and for expressing justice.^12^
